# The criteria used to rule out mild cognitive impairment impact dementia incidence rates in subjective cognitive decline

**DOI:** 10.1186/s13195-024-01516-6

**Published:** 2024-06-28

**Authors:** Tim Whitfield, Leonidas Chouliaras, Rachel Morrell, David Rubio, Darren Radford, Natalie L. Marchant, Zuzana Walker

**Affiliations:** 1 Division of Psychiatry, University College London, London, UK; 2 Department of Psychiatry, University of Cambridge School of Clinical Medicine, Cambridge, UK; 3 Specialist Dementia and Frailty Service, Essex Partnership University NHS Foundation Trust, St Margaret’s Hospital, Epping, UK

**Keywords:** Mild cognitive impairment, Subjective cognitive decline, Dementia, Incidence, Cognitive testing, Criteria

## Abstract

**Background:**

The research criteria for subjective cognitive decline (SCD) exclude mild cognitive impairment (MCI), but do not stipulate the use of specific MCI criteria. This study compared different approaches to defining (i.e., excluding) MCI during the ascertainment of SCD, focusing on the impact on dementia incidence rates in SCD.

**Methods:**

This cohort study utilized routine healthcare data collected in the Essex Memory Clinic from 1999 to 2023. Two different operationalizations of the SCD criteria were used to categorize the cohort into two SCD patient samples. One sample was based on local clinical practice – MCI was excluded according to the Winblad criteria (this sample was termed SCD_Winblad_). The other sample was created via the retrospective application of the Jak/Bondi criteria for the exclusion of MCI (termed SCD_Jak/Bondi_). Only patients aged ≥ 55 years at baseline with ≥ 12 months follow-up were considered for inclusion. The initial clinical/demographic characteristics of the samples were compared. Rates of incident dementia were calculated for each sample, and unadjusted and Mantel-Haenszel-adjusted incidence rate ratios were calculated to compare dementia incidence between the SCD samples.

**Results:**

The Essex Memory Clinic database included 2,233 patients in total. The SCD and study eligibility criteria were used to select SCD_Winblad_ (*n* = 86) and SCD_Jak/Bondi_ (*n* = 185) samples from the database. Median follow-up (3 years) did not differ between the two samples. The SCD_Jak/Bondi_ sample was significantly older than the SCD_Winblad_ at first assessment (median age: 74 versus 70 years) and had poorer scores on tests of global cognition, immediate and delayed verbal recall, and category fluency. Following adjustment for age, the dementia incidence rate ratio [95% confidence interval] was 3.7 [1.5 to 9.3], indicating a significantly greater rate of progression to dementia in SCD_Jak/Bondi_.

**Conclusions:**

This study highlights that the approach used to ascertain SCD has important implications for both SCD phenotypes and prognosis. This underscores the importance of how MCI is operationalized within SCD studies. More broadly, the findings add to a growing body of work indicating that objective cognition should not be overlooked in SCD, and offer a potential explanation for the heterogeneity across the SCD prognostic literature.

**Supplementary Information:**

The online version contains supplementary material available at 10.1186/s13195-024-01516-6.

## Background

Interest in the preclinical and prodromal stages of dementia continues to intensify within the research and medical communities. This trend is both motivated and typified by efforts to identify modifiable risk factors for dementia [1], as well as the recent emergence of disease-modifying immunotherapies for Alzheimer’s disease (AD) which target the mild dementia/mild cognitive impairment (MCI) stages [2].

Subjective cognitive difficulties in the absence of objective impairment on standardized tests are common in later life, affecting approximately 25% of the older population [3]. This phenomenon is clinically operationalized by the 2014 research criteria for subjective cognitive decline (SCD) [4]. Briefly, the criteria require both self-experienced decline in cognitive capacity, and normal performance on cognitive tests used to classify MCI and/or dementia [4].

Two meta-analyses [5, 6] and a multiple cohort analysis [7] found increased rates of incident dementia in people with SCD versus healthy controls. Nevertheless, individual prognosis is often uncertain [8], and there is significant heterogeneity in dementia incidence rates across the SCD literature [6]. A growing body of work has sought to identify individual- and study-level factors that may account for the variation in clinical outcomes in SCD [6, 7]. Wolfsgruber et al. [9] reported that, at a group level, SCD participants from the German Center for Neurodegenerative Diseases Longitudinal Cognitive Impairment and Dementia Study (DELCODE) had minor neuropsychological deficits. The same group later reported that, at the individual level, neuropsychological deficits are associated with an increased risk of incident MCI in SCD [10]. These studies highlight that, whilst (by definition) people with SCD lack objective cognitive impairment, some have subtle objective deficits, with these individuals having a worse prognosis.

Writing for the SCD Initiative (SCD-I), Molinuevo et al. [11] reviewed the literature to characterize how the SCD criteria are implemented in research settings, and to make recommendations for the future. The authors noted that studies differed in the way they operationalized the ‘MCI exclusion criterion’ within the SCD criteria. Some defined MCI according to conventional (i.e., Petersen [12] or Winblad [13]) criteria, which require only a single impaired cognitive score to be fulfilled. Others utilized the Jak/Bondi MCI criteria, which require more than one impaired score (either across or within cognitive domains), in order to capture a more ‘reliably impaired’ MCI phenotype, with a greater risk of progression to dementia [14]. The criteria used to define MCI effectively demarcate the maximum degree of cognitive deficits that can be present and still fulfil SCD criteria [11]. The upshot of this is that how MCI is defined during the ascertainment of SCD may have implications for SCD phenotypes/prognosis.

## Methods

### Reporting guidelines

This study is reported according to the REporting of studies Conducted using Observational Routinely-collected health Data (RECORD) guidelines [15]; see supplementary Table S4 for the RECORD checklist.

### Study design and setting

This study utilized routine healthcare data collected by the Essex Memory Clinic, a service operated by Essex Partnership University National Health Service Foundation Trust. The clinic provides in-depth assessment for patients presenting with cognitive symptoms. Clinical diagnosis is based on a consensus of professionals, including consultant old age psychiatrists and neuropsychologists. Assessment data (e.g., psychiatric/neuropsychological measures) are routinely recorded in a database to support healthcare provision and research.

### Study population

The version of the database used for this study contained data collected from April 1999 to August 2023, and included 2,233 patients. Two subsamples of these patients were included in analyses, based on the fulfilment of study eligibility criteria; given the current focus (i.e., dementia incidence), both samples only included patients aged ≥ 55 years at their initial assessment, with at least one follow-up visit ≥ 12 months later. For both samples, patients had to fulfil the research criteria for SCD [4] following their initial assessment. The criteria require both self-experienced persistent decline in cognitive capacity, and normal demographically-adjusted performance on standardized cognitive tests used to classify MCI/dementia. Importantly, different criteria were used to diagnose (i.e., exclude) MCI from each sample. For the first sample, MCI was diagnosed (i.e., excluded) according to the Winblad MCI criteria [13], while the Jak/Bondi criteria [14] were used to exclude MCI from the second sample (see ‘Derivation of SCD samples’ below). In-keeping with the SCD criteria, both samples excluded patients with currently elevated depressive/anxiety symptoms, and/or a history of bipolar disorder, schizoaffective disorder, schizophrenia, or alcohol use disorder. Both samples also excluded individuals with a history of stroke, head injury with loss of consciousness, epilepsy or neurodegenerative disease.

### Procedures

At patients’ initial assessment, a psychiatrist took a thorough medical, psychiatric and social history (including alcohol/tobacco use); and completed a neurological examination. Patients were also invited to undergo structural neuroimaging (MRI/CT) and blood screening for reversible causes of cognitive impairment. At both initial *and* follow-up assessments, a range of standardized psychiatric, neurological and medical measures, as well as a number of neuropsychological tests, were completed (see below); the medical history was also updated as applicable. Patients were asked to attend assessments with an informant where possible. For full details of clinical procedures, see Sadiq and colleagues [16].

### Measures

#### Affective symptoms

As noted above, in line with the SCD criteria, individuals with currently elevated affective symptoms were excluded from this study. Excluding these individuals minimizes the confounding effects of affective symptoms on subjective cognition [17]. Different measures were used to capture affective symptoms within the data collection period. Depressive and anxiety symptoms were initially captured using the Cornell Scale for Depression in Dementia (CSDD) [18], and the Rating Anxiety in Dementia (RAID) scale [19], respectively. These scales were subsequently replaced by the Hospital Anxiety and Depression Scale (HADS), comprising anxiety (HADS-A) and depression (HADS-D) subscales [20]. Patients exceeding published clinical cut-offs (i.e., CSDD ≥ 12; RAID ≥ 11; HADS-A/D ≥ 11) [18–20] were excluded.

#### Objective cognition

Global cognitive status was measured using the Mini-mental state examination (MMSE; scoring range: 0–30) [21] and the Cambridge Cognitive Examination-Revised (CAMCOG-R; scoring range: 0–105). Scores < 81 on CAMCOG-R are suggestive of dementia [22]. Episodic memory was measured using immediate and delayed recall scores from the Logical Memory (LM) subtest of the Wechsler Memory Scale-third [23] or -fourth [24] edition. LM scores were entered on the database as age-adjusted percentile scores. Psychomotor speed and executive task switching were assessed using parts A and B of the Trail-Making Test (TMT-A and -B), respectively. Scores were recorded as completion time in seconds (note: TMT trials were discontinued for patients making ≥ 2 errors). Verbal fluency was assessed using category fluency (total animals in 60 s) and letter fluency for ‘F’, ‘A’, and ‘S’ (for each letter, total correct words in 60 s). For the application of Jak/Bondi MCI criteria (see ‘Derivation of SCD samples’), raw cognitive scores had to be converted to *z*-scores. Category fluency, letter fluency, TMT-A and TMT-B raw scores were *z*-scored using age- and education-specific norms [25, 26]. LM data were originally recorded as age-corrected percentile scores; in line with previous work [27], LM immediate/delayed scores ≤ 16th percentile (corresponding to ≥ 1 SD below norms) were classified as impaired. Until 2012, premorbid IQ was estimated using the National Adult Reading Test [28]; thereafter it was measured using the Wechsler Test of Adult Reading [29]. During routine diagnostic work-up (i.e., using Winblad criteria – see ‘Diagnosis’), the multidisciplinary team considered the patient’s amount of formal education, estimated premorbid IQ, and premorbid occupational functioning; for patients with higher premorbid functioning, a stricter/higher threshold for cognitive impairment was utilized (the converse was true for individuals with lower premorbid functioning).

### Diagnosis

The criteria used to diagnose dementia varied over the data collection period, as criteria were superseded by updated versions. At present, dementia diagnosis is made according to the International Classification of Diseases (tenth revision) [30] in conjunction with dedicated criteria, including AD [31], vascular dementia (VaD) [32], dementia with Lewy bodies (DLB) [33] and frontotemporal dementia (FTD) [34]. For analyses, patients progressing to AD/AD with cerebrovascular disease (CVD) were combined into a single category (i.e., AD ± CVD). In the clinic, the term ‘subjective cognitive impairment’ (SCI) (see [35]) is used, rather than SCD – reflecting that the SCD criteria [4] were not yet published at the clinic’s inception. The entity of SCI closely aligns with SCD, although SCI is more inclusive (e.g., psychiatric disorders are not exclusionary, except where these are adjudged to fully account for cognitive symptoms). In the clinic, the diagnosis of MCI is made according to Winblad criteria [13] (summarized in Table 1). Prior to the publication of the Winblad criteria (i.e., from 1999 to 2004), MCI was defined as one or more impaired cognitive scores without functional impairment (a conception broadly similar to that encapsulated by the Winblad criteria). Patients who receive a diagnosis of MCI or SCI are invited for reassessment at 1 or 2 years, respectively.

**Table 1:** Diagnostic criteria used in this study to operationalize the ‘MCI exclusion criterion’ within the SCD research criteria

Winblad [13] MCI criteria used in the Essex Memory Clinic	Jak/Bondi [14] MCI criteria used for this study
1) Not normal, not demented (Does not meet criteria (DSM-IV, ICD-10) for a dementia syndrome) 2) Cognitive decline a. Self and/or informant report and impairment on objective cognitive tasks* and/or b. Evidence of decline over time on objective cognitive tasks 3) Preserved basic activities of daily living/minimal impairment in complex instrumental functions	1) General cognitive and functional performance sufficiently preserved such that a diagnosis of dementia cannot be made 2) Cognitive decline a. Both scores fall more than 1 SD below the age-specific normative mean within a cognitive domain; or b. One score falls more than 1 SD below the age-specific normative mean in all of the cognitive domains sampled

The Winblad criteria [13] are reproduced with permission from John Wiley and Sons. The Jak/Bondi criteria [14] are reproduced with permission from IOS Press. Abbreviations: MCI = mild cognitive impairment; DSM-IV = Diagnostic and Statistical Manual of Mental Disorders (4th edition); ICD-10 = International Statistical Classification of Diseases and Related Health Problems (10th Revision). *Whilst, for Winblad criteria, the clinic does not apply a rigid cut-off to classify MCI, cognitive scores falling below the 10th percentile (according to demographically-adjusted norms) are typically interpreted as impaired.

### Derivation of SCD samples

The derivation of the ‘first’ SCD sample essentially followed local clinical practice; only patients with SCI (see previous paragraph) who *also* fulfilled the SCD criteria [4] were included. For this sample, in line with routine practice, MCI was operationalized (excluded) according to Winblad criteria. For brevity, SCD diagnosed after ‘ruling out’ MCI according to Winblad criteria is hereafter denoted SCD_Winblad_ (for consistency, the same convention is used to denote patients who fulfil Winblad criteria, that is, MCI_Winblad_).

A ‘second’ SCD sample was created for this study, by retrospectively applying the SCD criteria to the assessment data of all patients originally diagnosed with SCI/MCI (see ‘Diagnosis’). Importantly, for this sample, Jak/Bondi [14] (rather than Winblad) criteria were used to diagnose/exclude MCI. Only patients who fulfilled SCD criteria [4] but did *not* fulfil Jak/Bondi MCI criteria were included in the second sample. This sample is hereafter termed SCD_Jak/Bondi_ (patients fulfilling Jak/Bondi criteria for MCI are hereafter termed MCI_Jak/Bondi_). Only patients originally categorized as SCI/MCI were considered for reclassification, because other diagnoses (e.g., dementia, psychiatric disorders) are excluded by SCD criteria. Importantly, the Winblad criteria require ‘self and/or informant report’ of cognitive dysfunction for the diagnosis of MCI. Thus, *self*-reported dysfunction (the defining feature of SCD) could not be assumed for patients with MCI_Winblad_. For these individuals, a case note review was undertaken; where self-reported difficulties could not be confirmed, patients were not eligible for reclassification as SCD_Jak/Bondi_.

Jak/Bondi MCI criteria (see Table 1) require cognitive tests to be categorized into domains. For the present study, each domain subsumed two tests/scores; the domains (constituents) were: verbal fluency (category fluency and letter fluency); episodic memory (LM immediate and delayed recall); and (after Bondi et al. [14]) psychomotor speed/executive function (TMT-A and -B). Patients are classified as MCI_Jak/Bondi_ if either: (1) *both* scores within a domain fall more than 1 SD below the demographically-adjusted normative means; or (2) at least one score in *all* domains falls more than 1 SD below the normative mean. Missing values in these six measures (including discontinued TMT trials) were coded as impaired, because missingness was associated with poorer CAMCOG-R scores (see supplementary Methods), and because this approach effectively excluded patients with an unclear cognitive profile from the SCD_Jak/Bondi_ sample.

### Statistical analyses

Descriptive and between-sample statistics were calculated for demographic, cognitive and follow-up characteristics. For cognitive variables used to operationalize Jak/Bondi criteria, *z*-scores are presented. For MMSE/CAMCOG-R, raw scores are reported. The proportion of missing data was also calculated and reported for each variable. To estimate the incidence rate of dementia, two new variables were calculated for each patient. The first was a binary event indicator (coded as 1/0 for patients who did/did not progress to dementia, respectively). A time-to-event (hereafter ‘follow-up’) variable was also calculated, capturing the time (in years) from initial assessment to diagnosis of dementia or end of follow-up (for patients who did not progress to dementia). For each sample, follow-up was summed across patients, corresponding to the total person-years at risk of dementia. The incidence rate of dementia was calculated for each sample by dividing the number of incident dementia cases by the total person-years at risk, and multiplying by 1,000. Incidence rates are thus interpretable as the number of new cases of dementia diagnosed amongst 1,000 patients with SCD each year [36].

Incidence rates of dementia were compared between samples via the incidence rate ratio, defined as the rate in SCD_Jak/Bondi_ divided by the rate in SCD_Winblad_. If between-sample differences were observed for any initial characteristic(s) (other than cognitive measures), this was treated as confounding and a Mantel-Haenszel adjusted incidence rate ratio was also calculated. For calculation, each sample is stratified according to the confounder, and the incidence rate is derived for each sample/stratum. Mantel-Haenszel methods are then used to combine the stratum-specific estimates [37]. The statistical significance of the unadjusted and Mantel-Haenszel adjusted incidence rate ratios is evaluated via a chi-squared test of the null hypothesis that the ratio is equal to 1.

Analyses were conducted in R 4.3.1 under RStudio 2023.12.1. The survival 3.5-7 package was used to calculate person-years at risk. The epi.2by2 function from the epiR 2.0.68 package was used to calculate the incidence rate for each sample, as well as the unadjusted and Mantel-Haenszel adjusted incidence rate ratios and 95% confidence intervals (CI). All statistical tests were two-sided and deemed significant at *p* < 0.05.

## Results

### Sample selection

**Fig. 1:**
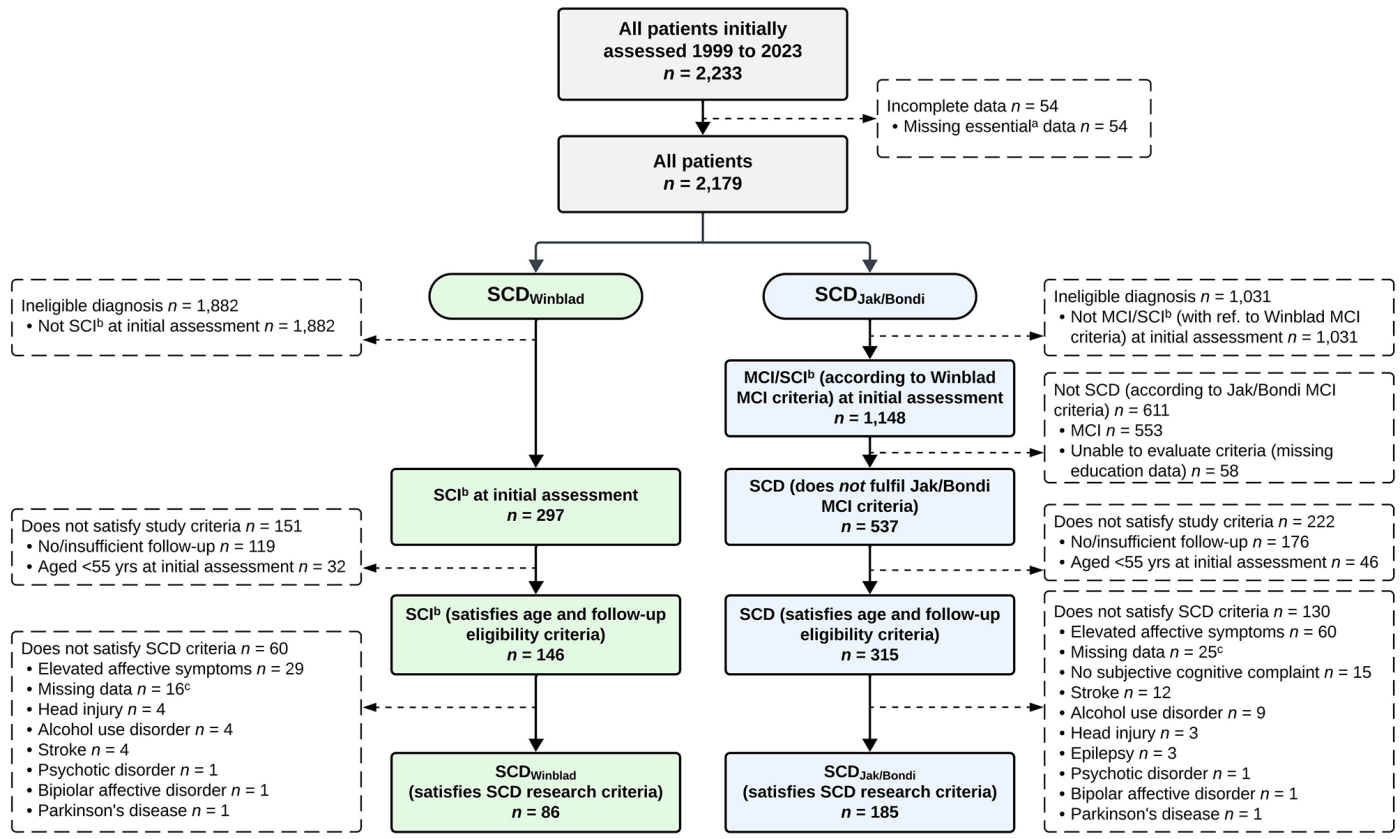
Flowchart detailing selection of SCD_Winblad_ and SCD_Jak/Bondi_ samples. Abbreviations: SCD = Subjective cognitive decline; SCI = Subjective cognitive impairment; RAID = Rating Anxiety in Dementia scale; CSDD = Cornell Scale for Depression in Dementia; HADS-A = Hospital Anxiety and Depression Scale-Anxiety subscale; HADS-D = Hospital Anxiety and Depression Scale-Depression subscale. ^a^Missing date of birth, visit date and/or diagnosis data. ^b^On the database, the term SCI, rather than SCD, is used (see Methods). ^c^Patients with missing data for measures of affective symptomatology (i.e., RAID/CSDD or HADS-A/HADS-D) or case notes were excluded, as it was not possible to determine their fulfilment of the SCD research criteria

See Fig. 1 for a flowchart outlining the derivation of the SCD_Winblad_ (*n* = 86) and SCD_Jak/Bondi_ (*n* = 185) samples.

### Sample initial characteristics and follow-up durations

Descriptive and comparative statistics for the samples’ demographic, cognitive and follow-up characteristics are presented in Table 2. There were no significant between-sample differences for sex, years of education, ethnicity, nor year of first visit. However, the median [interquartile range; IQR] age (years) of the SCD_Jak/Bondi_ sample (74.4 [66.5 to 80.1]) was greater than that of the SCD_Winblad_ sample (70.0 [61.7 to 74.8]; *p* < 0.01). For all *z*-scored cognitive tests, medians were > 0 for both groups, as would be expected for cognitively unimpaired samples. The samples significantly differed for CAMCOG-R, category fluency, LM immediate recall, and LM delayed recall (SCD_Winblad_ > SCD_Jak/Bondi_ in each case); no significant differences were observed for MMSE, TMT-A/B, nor letter fluency.

Whilst the median [IQR] number of visits was greater in the SCD_Jak/Bondi_ versus SCD_Winblad_ sample (3 [2 to 5], versus 3 [2 to 4]; *p* = 0.02), the length of follow-up (years) did not differ (3.2 [2.1 to 4.9], versus 3.4 [2.1 to 4.7]; *p* = 0.51).

**Table 2:** Sample demographic, cognitive and follow-up characteristics

	SCD_Winblad_ (*n* = 86)	SCD_Jak/Bondi_ (*n* = 185)	*p*
**Demographics**			
Age (years)	70.0 (61.7 to 74.8)	74.4 (66.5 to 80.1)	**< 0.01**
Sex (female)	39 (45%)	76 (41%)	0.60
Education (years) Ethnicity*	11 (10 to 13)	11 (10 to 12)	0.09
White Asian Mixed	82 (97%) 1 (1%) 2 (2%)	177 (95%) 5 (3%) 2 (1%)	
Black	0 (0%)	1 (1%)	0.48
**Observation period**			
Year of first visit 2000–2009 2010–2019 2020–	35 (41%) 50 (58%) 1 (1%)	80 (43%) 102 (55%) 3 (2%)	0.88
Visits (*n*)	3 (2 to 4)	3 (2 to 5)	**0.02**
Follow-up (years)	3.4 (2.1 to 4.7)	3.2 (2.1 to 4.9)	0.51
**Cognitive measures**			
MMSE (total)	29 (28 to 30)	28 (27 to 29)	0.06
CAMCOG-R (total)	97 (94 to 99)	94 (91 to 98)	**< 0.01**
TMT-A (*z*-score)	0.1 (-0.6 to 0.9)	0.4 (-0.3 to 0.9)	0.18
TMT-B (*z-*score)	0.9 (0.4 to 1.3)	0.7 (0.1 to 1.3)	0.26
Category fluency (*z-*score)	0.9 (-0.2 to 1.8)	0.3 (-0.4 to 1.3)	**0.02**
Letter fluency (*z-*score)	0.2 (-0.3 to 0.9)	0.2 (-0.3 to 1.0)	0.99
LM immediate (*z-*score)	0.7 (-0.3 to 1.3)	0.3 (-0.3 to 1.0)	**0.03**
LM delayed (*z-*score)	0.7 (0.3 to 1.3)	0.3 (-0.7 to 1.0)	**< 0.01**

Continuous variables are median (IQR); categorical variables are *n* (%). Inferential statistics are from chi-squared/Fisher’s exact or Mann-Whitney *U* tests (**bold ***p*-values are < 0.05). Abbreviations: SCD = subjective cognitive decline; MMSE = Mini-Mental State Examination; CAMCOG-R = Cambridge Cognitive Examination-Revised; TMT-A = Trail-Making Test part A; TMT-B = Trail-Making Test part B; LM = Logical Memory; IQR = interquartile range. *Ethnicity data for one patient from the SCD_Winblad_ sample were missing. The ethnicity categories map to those employed in the UK Census: White (White); Asian or Asian British (Asian); Mixed or multiple ethnic groups (Mixed); and Black, Black British, Caribbean or African (Black).

### Data cleaning and missing data

Prior to analysis, cognitive variables were checked for values falling outside the valid scoring range; aberrant values were corrected by referring back to case notes. Where this did not satisfactorily resolve queries, values were deleted. For the missingness proportions in each variable/sample, see supplementary Table S1. For both samples, missingness was ≤ 5% for all variables.

### Overlap between samples

A total of 74 patients were common to both SCD samples (these individuals had SCD irrespective of the criteria used to exclude MCI). Twelve patients in the SCD_Winblad_ sample were reclassified as MCI under Jak/Bondi criteria. The SCD_Jak/Bondi_ sample included the remaining 74 patients from the SCD_Winblad_ sample, as well as 111 patients originally diagnosed with MCI_Winblad_.

### Incidence rates of dementia

Of the 86 patients in the SCD_Winblad_ sample, 5 (6%) progressed to dementia (3 to AD ± CVD, 1 to DLB, and 1 to VaD; see Table 3) during 335 person-years of observation. The incidence rate [95% CI] of dementia was 14.9 [4.9 to 34.9] per 1,000 person-years.

**Table 3:** Diagnostic outcomes at follow-up

Outcome	SCD_Winblad_ (*n* = 86)	SCD_Jak/Bondi_ (*n* = 185)
**Did not progress to dementia** ^a^ SCD MCI^b^	81 (94.2%) 55 (64.0%) 26 (30.2%)	133 (71.9%) 113 (61.0%) 20 (10.9%)
**Progressed to dementia** AD ± CVD DLB VaD FTD Unspecified dementia	5 (5.8%) 3 (3.4%) 1 (1.2%) 1 (1.2%) 0 (0.0%) 0 (0.0%)	52 (28.1%) 41 (22.2%) 5 (2.7%) 3 (1.6%) 2 (1.1%) 1 (0.5%)

Abbreviations: SCD = subjective cognitive decline; MCI = mild cognitive impairment; AD ± CVD = Alzheimer’s disease dementia with or without cerebrovascular disease; DLB = dementia with Lewy bodies; VaD = vascular dementia; FTD = frontotemporal dementia. ^a^In the SCD_Winblad_ sample, two patients were diagnosed with Parkinson’s disease (PD) during follow-up; one of these individuals remained SCD while the other (also) progressed to MCI. In the SCD_Jak/Bondi_ sample, three patients were diagnosed with PD during follow-up; one of these individuals remained SCD while the other two (also) progressed to MCI. ^b^The criteria used to diagnose MCI corresponded to those used to operationalize the SCD criteria.

Of the 185 patients in the SCD_Jak/Bondi_ sample, 52 (28%) progressed to dementia (41 to AD ± CVD, 5 to DLB, 3 to VaD, 2 to FTD, and 1 to unspecified dementia) during 689 person-years of observation. The incidence rate [95% CI] of dementia was 75.5 [56.4 to 98.9] per 1,000 person-years.

### Incidence rate ratio

The unadjusted incidence rate ratio [95% CI] was 5.1 [2.0 to 16.2], indicating that the incidence rate of dementia was greater (*p* < 0.01) in the SCD_Jak/Bondi_ (versus SCD_Winblad_) sample. However, the SCD_Jak/Bondi_ sample was on average 4.4 years older than the SCD_Winblad_ sample (median 74.4 versus 70.0 years; *p* < 0.01). This difference was a confound, as dementia risk increases with age [38]. To derive an adjusted estimate, both samples were age-stratified, and incidence rate ratios calculated for each pair of strata (see Table 4); estimates were then combined using Mantel-Haenszel methods. Following Mantel-Haenszel adjustment for age, the overall ratio was nominally attenuated (3.7 [1.5 to 9.3]), but continued to indicate a greater dementia incidence rate in SCD_Jak/Bondi_ (*p* < 0.01).

**Table 4:** Overall and age strata-specific incidence rate ratios

Stratum/sample*	*n* events / total	Age (years)		Incidence rate ratio	
		Median (IQR)	*p*	Estimate [95% CI]	*p*
*55 to 65 years*					
SCD_Winblad_ SCD_Jak/Bondi_	1 / 30 4 / 43	59.5 (57.6 to 61.8) 61.5 (59.2 to 63.3)	0.09	2.7 [0.3 to 134.5]	0.64
*65 to 75 years*					
SCD_Winblad_ SCD_Jak/Bondi_	1 / 36 10 / 54	71.4 (68.0 to 73.6) 71.6 (68.7 to 73.6)	0.76	6.4 [0.9 to 277.9]	0.09
*75 years and above*					
SCD_Winblad_ SCD_Jak/Bondi_	3 / 20 38 / 88	78.4 (76.7 to 83.3) 80.2 (77.2 to 83.1)	0.64	3.2 [1.0 to 16.4]	0.07
		**Overall (unadjusted)**:		5.1 [2.0 to 16.2]	**< 0.01**
		**Overall (age-adjusted)**:		3.7 [1.5 to 9.3]	**< 0.01**

Inferential statistics are from Mann-Whitney *U* tests (for comparing age between samples/strata) or chi-squared tests (for assessing the significance of incidence rate ratios; **bold ***p*-values are < 0.05). The total person-years at risk for each SCD_Winblad_ stratum were: 55–65 yrs = 116; 65–75 yrs = 143; and ≥ 75 yrs = 76. The total person-years at risk for each SCD_Jak/Bondi_ stratum were: 55–65 yrs = 170; 65–75 yrs = 223; and ≥ 75 yrs = 296. Abbreviations: SCD = subjective cognitive decline; IQR = interquartile range; CI = confidence interval. *Lower bounds for age strata are inclusive, upper bounds (where applicable) are exclusive.

### Proportion progressing to MCI

The proportion of patients with a ‘final’ diagnosis of MCI was greater in the SCD_Winblad_ versus SCD_Jak/Bondi_ sample (30% versus 11%; *p* < 0.01).

## Discussion

This study harnessed routinely-collected memory clinic data to define two SCD samples according to different operationalizations of the Jessen et al. research criteria [4], and compared the incidence rate of dementia between them. The main finding was that the age-corrected incidence rate of dementia was over threefold greater in SCD patients when the Jak/Bondi (versus Winblad) criteria were used to exclude MCI.

Interestingly, the SCD_Jak/Bondi_ sample (*n* = 185) was larger than the SCD_Winblad_ (*n* = 86) sample. The retrospective application of the Jak/Bondi criteria resulted in 111 patients originally diagnosed with MCI (according to Winblad criteria) being reclassified as SCD. Whilst some patients were reclassified from SCD_Winblad_ to MCI_Jak/Bondi_, these were fewer in number (*n* = 12). Interestingly, none of these 12 individuals progressed to dementia during follow-up. Whilst the increased dementia incidence rate in SCD_Jak/Bondi_ thus appears to be driven by the inclusion of patients formerly classified as MCI_Winblad_, it may also reflect that 12 patients who did not progress to dementia were included in the SCD_Winblad_ but not the SCD_Jak/Bondi_ sample. Importantly, the SCD_Jak/Bondi_ sample was older than the SCD_Winblad_ sample. Dementia risk increases with age [38] – it is thus likely that, by broadening the definition of SCD to include individuals with worse cognition, a greater number of older adults with incipient cognitive decline were captured.

The SCD_Jak/Bondi_ (versus SCD_Winblad_) sample had lower baseline scores on the CAMCOG-R, category fluency, and both immediate and delayed LM recall. A previous study of cognitively unimpaired participants found that scores on the 5-item preclinical Alzheimer’s cognitive composite (PACC5) – which measures global cognition, category fluency, verbal memory and executive function – are inversely associated with cerebral amyloid-β load [39]. Moreover, an earlier study from the Essex Memory Clinic found that poorer global cognition and delayed verbal recall predicted incident AD in a combined SCD/MCI sample [40]. The present finding that, versus SCD_Winblad_, SCD_Jak/Bondi_ is characterized by poorer global cognition, category fluency and verbal recall, as well as an increased risk of dementia (predominantly due to AD) is thus broadly in-keeping with prior work linking specific cognitive profiles to early AD.

We observed dementia incidence rates of 15 and 76 per 1,000 person-years in SCD_Winblad_ and SCD_Jak/Bondi_, respectively. Slot et al. [7] leveraged six memory clinic cohorts to estimate the incidence rate of dementia in 1,530 patients with SCD (mean ± SD age: 67 ± 9 years); five of the cohorts excluded MCI on the basis of a single impaired score, while the remaining cohort excluded MCI via Jak/Bondi criteria. Pooling across cohorts, Slot et al. reported a dementia incidence rate of 20 per 1,000 person-years. In a study using National Alzheimer’s Coordinating Center (NACC) data, Ward et al. [41] calculated that the incidence rate of dementia was 134 per 1,000 person-years in 3,428 patients with MCI (age: 76 ± 7 years) diagnosed on the basis of a single impaired score. The incidence rate in the current SCD_Winblad_ sample thus appears broadly comparable to that observed in the multicenter study by Slot et al. [7] (which largely employed a similar approach to excluding MCI). The incidence rate in the SCD_Jak/Bondi_ sample is intermediate between Slot et al. and the MCI sample from NACC [41]. Given the SCD_Jak/Bondi_ sample comprised 74 patients originally classified as SCD_Winblad_, plus 111 reclassified from MCI_Winblad_, this intermediate incidence rate appears plausible. Moreover, a previous study by Rhodius-Meester et al. [42] reported prognostic data for three SCD samples, one of which was ascertained by the German Dementia Competence Network (DCN; *n* = 269). Importantly, the DCN use Jak/Bondi criteria for MCI [43]; their SCD operationalization thus appears analogous to SCD_Jak/Bondi_. Rhodius-Meester et al. [42] did not report dementia incidence rates, but they did include sufficient data to calculate these – we calculated a rate of 54 per 1,000 years for the DCN sample. This rate appears lower than that observed for SCD_Jak/Bondi_ in the current study (76 per 1,000 years); however, the current patients were older than the DCN sample (age: 73 ± 9 versus 66 ± 8 years). Whilst the incidence rate of dementia in SCD_Jak/Bondi_ may thus be higher than expected for an SCD sample, it remains broadly congruent with findings from other cohorts.

Whilst fewer patients developed dementia in the SCD_Winblad_ (versus SCD_Jak/Bondi_) sample, a *greater* proportion had MCI at their last available assessment (30% versus 11%). Whilst this may caution against conceptualizing SCD_Winblad_ as a reliably ‘benign’ or ‘stable’ phenotype, the between-sample difference in MCI incidence does not affect the overall conclusions of this study; the majority of SCD_Jak/Bondi_ patients who progressed to dementia would likely have transitioned through the MCI stage (not reflected in the ‘final’ proportions with MCI reported above). Moreover, MCI does not always progress to dementia [44], and can revert (i.e., improve) in around 8% of cases in clinical settings [45]. Thus, whilst a greater proportion of patients with SCD_Winblad_ progressed to MCI, overall this phenotype had a markedly better prognosis (i.e., a lower dementia incidence rate) versus SCD_Jak/Bondi_. One question not answerable using the current design is whether there is a difference in the rate of cognitive decline between SCD_Winblad_ and SCD_Jak/Bondi_; this could be a fruitful line of investigation for future research.

Recall that Jak/Bondi criteria employ a conservative cut-off to classify cognitive scores as impaired (-1 SD below the normative mean), but that more than one impaired score is required for MCI (see Methods/Table 1). Bondi et al. [14] retrospectively applied the Jak/Bondi MCI criteria to a sample of Alzheimer’s Disease Neuroimaging Initiative participants, including 846 patients with MCI_Winblad_, and 304 cognitively unimpaired individuals (without SCD). Following reclassification, 401 participants had MCI_Jak/Bondi_, while 749 were cognitively unimpaired. Compared to the original MCI_Winblad_ sample, MCI_Jak/Bondi_ was characterized by more consistent cognitive impairment; a greater proportion of *APOE* ε4 carriers; more AD-like CSF profiles; and a greater dementia incidence rate. In the present study, the application of Jak/Bondi criteria similarly classified a smaller number of individuals as MCI (here resulting in a greater number with SCD). In summary, Jak/Bondi criteria take a more conservative (versus Winblad) neuropsychological approach to defining MCI, resulting in fewer, more impaired ‘cases’ of MCI, with a greater risk of dementia (i.e., more specific for predicting progression). Conversely, Jak/Bondi criteria may be more prone to miss subtle cognitive deficits (i.e., less sensitive for predicting progression [46]). The use of Jak/Bondi criteria to rule out MCI thus appears to capture an SCD phenotype with worse objective cognition/prognosis.

Whilst Jak/Bondi and Winblad criteria take different approaches to operationalizing MCI, they are not mutually exclusive, resulting in a degree of ‘overlap’ between the current SCD samples. An alternative would be to categorize patients into three non-overlapping groups: those with SCD irrespective of MCI criteria (SCD_Winblad_/SCD_Jak/Bondi_), and those with SCD under one criteria but MCI under the other (i.e., SCD_Winblad_/MCI_Jak/Bondi_ and MCI_Winblad_/SCD_Jak/Bondi_). This approach may improve prognostic predictions. Unfortunately, this study lacked statistical power to investigate this empirically. In any case, this may be a primarily research-oriented question, as most clinical settings only utilize one type of MCI criteria.

The prognostic implications of different approaches to excluding MCI during the ascertainment of SCD have previously been discussed [11, 43], but empirical data have been lacking. Nevertheless, there is increasing recognition that SCD may not be synonymous with entirely ‘normal’ objective cognition, and that minor neuropsychological deficits have prognostic value in SCD. Using DELCODE data, Wolfsgruber et al. [9] demonstrated that, at the group level, patients with SCD have minor neuropsychological deficits (approximately 0.25–0.5 SD in magnitude) versus controls. The same group recently showed that SCD patients with (versus without) minor neuropsychological deficits had a faster cognitive decline and increased risk of MCI [10]. The DELCODE investigators operationalized MCI as a deficit of at least 1.5 SD on any test – an approach comparable to that used for the SCD_Winblad_ sample in the current study. In spite that the SCD sample in DELCODE most closely aligns with the sample with better cognition/prognosis in the current study, those DELCODE SCD participants with subtle cognitive deficits continued to have a worse prognosis. In summary, despite that objective cognition is unimpaired in SCD, variation in scores (comfortably within the normal range) is linked to prognosis, both at the between-individual and between-sample levels. Whilst this suggests that the neuropsychological cut-offs employed by MCI criteria miss subtle – yet prognostically meaningful – cognitive deficits, more sensitive/thorough tests may be required to capture them, which are not available in all clinical settings.

The finding that excluding MCI via more stringent criteria yielded an SCD sample with worse cognition/prognosis is, arguably, unsurprising. Nevertheless, this finding remains important; there is significant heterogeneity in dementia incidence rates across the SCD literature [5, 47], and recent critiques have questioned the prognostic value of an SCD ‘diagnosis’ [48, 49]. Attempts to explain the heterogeneous dementia incidence rates in SCD have generally been unsuccessful. A recent meta-analysis evaluated numerous potential moderators of dementia incidence in SCD studies (including how SCD is defined, demographic/genetic factors, recruitment source, and follow-up duration) [6], but no significant moderators were identified (note: statistical power may have been lacking). Interestingly, neither the type of criteria used to exclude MCI, nor objective cognition more generally, were explored as candidate moderators. Whilst, in clinical practice, prognostic evaluation is primarily informed by individual patient characteristics, the present work suggests that the particular approach used to define MCI in a given clinical setting has important prognostic implications for individuals with SCD [4]. 

Reviewing the operationalization of SCD in research settings for the SCD-I, Molinuevo et al. [11] encouraged greater harmonization of SCD characterization across studies, to facilitate comparisons and evidence synthesis. Nevertheless, the authors acknowledged that variation in how SCD is defined has advanced scientific understanding in the field. Indeed, the authors did *not* recommend specific MCI criteria for SCD studies, because: the SCD/MCI boundary may not be clearcut, neuropsychological batteries vary across settings, and the choice of ‘liberal’ or ‘conservative’ MCI criteria depends on research questions. We echo the recommendations of the SCD-I: the optimum approach taken to exclude MCI in future SCD studies will likely depend on the research question. For example, in clinical trials targeting AD, the Jak/Bondi criteria may be optimal for excluding MCI (in order to ‘enrich’ the sample for preclinical AD and maximize statistical power [50]).

### Strengths

The current work has a number of strengths. The present use of routinely-collected clinical data is no doubt a strength, given dementia risk is elevated in clinical, but not community-dwelling SCD populations [7]. This also increases the generalizability of the work to settings which assess and provide prognosis to individuals with cognitive symptoms. Moreover, all patients in the study underwent a thorough medical, neurological and psychiatric evaluation, and were assessed using a range of neuropsychological tests. This thorough characterization enabled the derivation of two samples fulfilling SCD criteria, differing only in the neuropsychological criteria used to operationalize (i.e., exclude) MCI. Furthermore, patients were followed-up for an average of 3 years, enabling dementia incidence rates to be estimated. The diagnosis of dementia (including subtype) was made by consensus, which improves diagnostic accuracy compared to individual clinicians [51]. Lastly, the calculation of the incidence rate ratio made use of Mantel-Haenszel methods which enabled adjustment for the confounding effect of age.

### Limitations

This study also has limitations. To derive adequate samples of SCD patients, we used all available data (collected over three decades). Jutten et al. [52] discuss how ‘average performance’ on cognitive tests can drift over time, rendering norms outdated. Our reclassification of patients according to ‘fixed’ cognitive norms may thus have delineated subtly different SCD phenotypes at different times (although the temporal distribution of assessments appeared comparable between samples – see Table 2). This limitation partly reflects that the data were collected during routine healthcare at a single center [15]. Efforts to replicate the current findings in data collected within tighter timeframes (perhaps using prospective multicenter designs) are thus welcomed. The administration of TMT in this study differed from the typical procedure – here, patients were discontinued at two errors, whereas in other settings, assessors correct all errors without discontinuing patients (though the potential impact of this on results appears limited; see supplementary Discussion note 1). The MCI criteria utilized in this study vary in the precision with which they define cognitive impairment. The Winblad criteria require ‘impairment on objective cognitive tasks’, while the Jak/Bondi criteria feature specific cutoffs according to population norms. Both samples had a very high proportion of white individuals; whilst this is in-keeping with the characteristics of the population served by the memory clinic (see supplementary Discussion note 2), further research in samples with better representation of other ethnic groups will be required to assess the extent to which the current findings generalize in other populations. Further, the normative data used to calculate *z*-scores for each domain did not adjust for identical demographic factors; for verbal fluency and psychomotor speed/executive function, age- and education-adjusted norms were used, whereas the available norms for episodic memory only adjusted for age. Nor did we have access to ethnically and culturally appropriate test norms; a previous study reported that using ‘combined’ ethnicity norms can result in misclassifying scores for some groups [53]. In the interests of standardization and replicability, future SCD investigators are encouraged to characterize samples using ethnically and educationally appropriate norms, and to employ MCI criteria with precise cutoffs. Furthermore, the available tests permitted only three cognitive domains to be operationalized for Jak/Bondi criteria; given one of the ‘routes’ to diagnosis of MCI_Jak/Bondi_ is one impaired score within each assessed domain, this could have led to overdiagnosis of MCI, though Bondi et al. [14] also utilized three cognitive domains in their validation study. Considering the specific measures used to characterize cognitive domains, the tests used by Bondi et al. [14] for the language and episodic memory domains appeared less correlated than in the present study (see supplementary Discussion note 3). This could have resulted in a degree of diagnostic misclassification in the current study; future investigators are encouraged to utilize a greater number/range of cognitive domains, as well as sufficiently independent constituent tests, in order to mitigate this possibility. The defining characteristic of SCD is a self-experienced decline in cognitive capacity [4]. A retrospective case note review was required to ascertain the presence/absence of this feature for some patients, and this could not be determined in a small number, resulting in their exclusion. More generally, we did not have access to a standardized measure of subjective cognition (e.g., the SCD interview [54]), which precluded a comparison of subjective cognitive profiles between samples. This could be a fruitful topic for future investigations. Lastly, there is increasing interest in the role of biomarkers for prognostication in SCD [55]. Unfortunately, a lack of comprehensive biomarker data prevented the characterization of SCD according to (e.g.) the ATN framework [56]; we recommend that future projects include biomarker data where possible.

## Conclusions

In conclusion, this study harnessed routine healthcare data to evaluate the prognostic implications of alternative operationalizations of the SCD research criteria. Compared to SCD_Winblad_, the SCD_Jak/Bondi_ sample was larger, older and had worse initial objective cognition. Following adjustment for age, the rate of incident dementia was over threefold greater in SCD_Jak/Bondi_ versus SCD_Winblad_. The present work adds to the literature highlighting the prognostic utility of objective cognition in individuals with SCD. This may partially account for the heterogeneity in the SCD prognostic literature [6], and facilitate wider efforts to better characterize and standardize approaches to defining SCD [11].

### Electronic supplementary material

Below is the link to the electronic supplementary material.

Supplementary Material 1

## Data Availability

The data underpinning this study were acquired during routine healthcare, and the patients did not consent to external data sharing. Moreover, the HRA has not approved external data sharing.

## Abbreviations

AD: Alzheimer’s disease
AD ± CVD: Alzheimer’s disease with or without cerebrovascular disease
ATN: Amyloid, Tau and Neurodegeneration
CAMCOG-R: Cambridge Cognitive Examination-Revised
CI: Confidence interval
CODEC: Predictors of COgnitive DECline in attenders of memory clinic
CSDD: Cornell Scale for Depression in Dementia
DELCODE: German Center for Neurodegenerative Diseases Longitudinal Cognitive Impairment and Dementia Study
DLB: Dementia with Lewy bodies
FTD: Frontotemporal dementia
HADS: Hospital Anxiety and Depression Scale
HADS-A: Hospital Anxiety and Depression Scale-anxiety subscale
HADS-D: Hospital Anxiety and Depression Scale-depression subscale
HRA: Health Research Authority
IQR: Interquartile range
LM: Logical Memory
MCI: Mild cognitive impairment
MMSE: Mini-mental state examination
NACC: National Alzheimer’s Coordinating Center
PACC5: 5-item preclinical Alzheimer’s cognitive composite
RAID: Rating Anxiety in Dementia scale
RECORD: REporting of studies Conducted using Observational Routinely-collected health Data
SCD: Subjective cognitive decline
SCD-I: Subjective cognitive decline-Initiative
SCI: Subjective cognitive impairment
TMT: Trail-Making Test
TMT-A: Trail-Making Test part A
TMT-B: Trail-Making Test part B
VaD: Vascular dementia
