# Supplementary material for: The criteria used to rule out mild cognitive impairment impact dementia incidence rates in subjective cognitive decline
Source: Alzheimers Res Ther. 2024 Jun 28;16:142. doi: 10.1186/s13195-024-01516-6 (PMC11212190; doi:10.1186/s13195-024-01516-6)
Supplement: Supplementary file 1 — Supplementary Material 1 [file 13195_2024_1516_MOESM1_ESM.docx]

**Manuscript title:** The criteria used to rule out mild cognitive impairment impact dementia incidence rates in subjective cognitive decline

**Supplementary Methods**

Under the ‘Derivation of SCD samples’ subsection of the main text Methods, it states ‘Missing values in [the] six measures [used to classify patients according to Jak/Bondi MCI criteria] were coded as impaired, because missingness was associated with poorer CAMCOG-R scores […]’.

This statement relates to the 1,148 patients who were ‘originally’ diagnosed with MCI or SCI according to Winblad MCI criteria (see the first flowchart box under the ‘SCD_Jak/Bondi_’ subsample in main text Figure 1). Fifty-eight of these patients lacked education data, and were therefore not classifiable according to Jak/Bondi criteria (note: two of the three sets of norms we used were adjusted for education). Of the remaining 1,090 patients, 295 (27%) had one or more missing values for the six cognitive tests used to operationalize the Jak/Bondi MCI criteria (i.e., LM immediate, LM delayed, TMT-A, TMT-B, letter fluency, category fluency), and 795 (73%) had no missing values for these variables.

A non-parametric t-test revealed that the median CAMCOG-R total score was greater in the 795 patients with no missing cognitive scores, compared to the 295 patients with one or more missing cognitive scores (medians = 92 versus 87; *p* <0.01).

**Supplementary Results**

**Table S1.** Missing data summary

| **Variable** | **SCD_Winblad_ (*n* = 86)** | **SCD_Jak/Bondi_ (*n* = 185)** |
| --- | --- | --- |
| RAID | 63 (73%) | 121 (65%) |
| CSDD | 63 (73%) | 121 (65%) |
| HADS-A | 17 (20%) | 52 (28%) |
| HADS-D | 17 (20%) | 52 (28%) |
| TMT-A (*z*-score) | 1 (1%) | 0 (0%) |
| Category fluency (*z-*score) | 1 (1%) | 0 (0%) |
| Letter fluency (*z-*score) | 1 (1%) | 0 (0%) |
| Education (years) | 1 (1%) | 0 (0%) |
| TMT-B (*z* score) | 1 (1%) | 0 (0%) |
| MMSE (total) | 1 (1%) | 0 (0%) |
| CAMCOG-R (total) | 1 (1%) | 0 (0%) |
| LM immediate (*z* score) | 1 (1%) | 0 (0%) |
| Ethnicity | 1 (1%) | 0 (0%) |
| LM delayed (*z* score) | 0 (0%) | 0 (0%) |
| Age (years) | 0 (0%) | 0 (0%) |
| Sex | 0 (0%) | 0 (0%) |

Data are *n* (%) missing for each variable. Please note: in the main text, the Methods contains the statement ‘For both samples, missingness was ≤5% for all variables.’ Whilst higher missingness proportions are reported for the uppermost 4 variables above, this reflects that the measurement of both depressive and anxiety symptoms was changed throughout the study. Thus, the missingness for ‘the psychoaffective measure in use at a given time’ remained ≤5%. Data are *n* (%) and ordered in decreasing proportions of missingness. Abbreviations: RAID = Rating Anxiety in Dementia; CSDD = Cornell Scale for Depression in Dementia; HADS-A = Hospital Anxiety and Depression Scale-anxiety subscale; HADS-D = Hospital Anxiety and Depression Scale-depression subscale; SCD = subjective cognitive decline; MMSE = Mini-Mental State Examination; CAMCOG-R = Cambridge Cognitive Examination-Revised; TMT-A = Trail Making Test part A; TMT-B = Trail Making Test part B; LM = Logical Memory.

**Supplementary Discussion**

**Note 1**

Under the ‘Limitations’ subsection of the main text Discussion, it states ‘The administration of TMT [Trail-making test] in this study differed from the typical procedure – here, patients were discontinued at two errors, whereas in other settings, assessors correct all errors without discontinuing patients (the potential impact of this on results appears limited; see supplementary Discussion note 1)’. We expand on and explore the implications of this statement below:

Here we consider how the TMT administration procedure used in this clinical study could affect results. The primary potential impact could be that discontinued TMT trials could result in some patients being excluded from the study, who would otherwise have been included under conventional TMT rules. The potential impact of the current procedure on the ascertainment of the SCD_Winblad_ sample appears modest, because discontinued TMT trials do not, in isolation, rule out a ‘diagnosis’ of SCD_Winblad_ in the clinic. The potential impact on the derivation of the SCD_Jak/Bondi_ sample may be greater – discontinued TMT trials were classified as impaired for applying the Jak/Bondi criteria. We identified cases where a discontinued TMT trial may have resulted (note: we state ‘may have resulted’, because even if these trials had been allowed to continue, they may still have resulted in an impaired completion time) in a patient being excluded from the SCD_Jak/Bondi_ sample (i.e., due to fulfilling MCI_Jak/Bondi_). None of the patients who had SCD under Winblad but MCI under Jak/Bondi criteria (*n* = 12) were potentially excluded due to discontinued TMT trials. Of the 236 patients excluded from SCD_Jak/Bondi_ due to having MCI (all of whom also had MCI under Winblad criteria), 23 may have been classified as MCI_Jak/Bondi_ as a consequence of having a TMT-A and/or TMT-B trial discontinued. These 23 did not include patients for whom both *z*-scores within the episodic memory and/or verbal fluency domains were impaired, because these individuals would have been excluded irrespective of the TMT administration procedure. In 1 of these 23 individuals, discontinuation of TMT-B (may have) resulted in the patient having one impaired score in each of the three domains (they thus fulfilled Jak/Bondi criteria for MCI). In 21 of these 23 patients, TMT-A completion time (which was *not* discontinued) was impaired, and TMT-B was discontinued; these patients were excluded due to having two impaired TMT scores. In the final patient, both TMT-A and TMT-B were discontinued – this patient was excluded accordingly.

In unplanned sensitivity analyses, we added these 23 individuals to the SCD_Jak/Bondi_ sample (under the ‘worse case’ assumption that all of them would have been classified as SCD_Jak/Bondi_ had they not had one or more TMT trials discontinued at two errors) and recalculated the incidence rate ratio; the ‘new’ sample sizes were: SCD_Winblad_ (*n* = 86) and SCD_Jak/Bondi_ (*n* = 208). The revised incidence rate ratios [95% CI] were: unadjusted 6.1 [2.5 to 19.3]; and Mantel-Haenszel age-adjusted 4.5 [1.8 to 11.2] (both *p* <0.01). These sensitivity analyses suggest that any impact of the local (versus typical) TMT administration procedure would have biased the incidence rate ratio towards the null (i.e., potentially underestimating it), meaning that the observed difference in dementia incidence between SCD operationalizations appears robust.

**Note 2**

Under the ‘Limitations’ subsection of the main text Discussion, it states ‘Both samples had a very high proportion of white individuals; whilst this is in-keeping with the characteristics of the population served by the memory clinic […]’. Please see Table S2 for the ethnicity characteristics of: each SCD sample (taken from Table 2 in the main text); the 2011 UK Census; and the 2021 UK Census. These data are provided to enable a broad comparison of the ethnicity characteristics of the study patients versus the local population served by the clinic, in order to ascertain whether the mechanisms that generated the current data led to an underrepresentation (versus the local population) of individuals from Asian, black, mixed, and other ethnic groups.

**Note 3**

Under the ‘Limitations’ subsection of the main text Discussion, it states ‘Considering the specific measures used to characterize cognitive domains, the tests used by Bondi et al. for the language and episodic memory domains appeared less correlated than in the present study […]’. We expand on and explore the implications of this statement below (here, all references to MCI criteria refer to Jak/Bondi criteria unless specified otherwise):

The Jak/Bondi criteria (1) operationalize MCI as (i) two scores falling more than 1 SD below the age-specific normative mean within a cognitive domain; or (ii) one score falling more than 1 SD below the age-specific normative mean in all of the cognitive domains sampled (see also Table 1 in the main text). In the validation study, Bondi et al. (1) argue that this approach captures a more ‘reliable’ MCI phenotype – that is, defined by more reliable cognitive impairment versus the ‘single impaired score’ approach of the Winblad (2) and Petersen (3) criteria. This rationale has implications for the tests selected to constitute a ‘cognitive domain’. That is, whilst each of the two tests needs to measure a related cognitive ability (in order to justify their inclusion within a single cognitive domain), they should not be so closely related that they effectively measure the same thing – otherwise, the rationale of the criteria to define a reliably impaired MCI phenotype could be undermined. We compared the tests used to define cognitive domains in Bondi et al. and the present study, paying close attention to their statistical relatedness (judged using our own or external data; see Table S3 for details). Both Bondi et al. and the present study defined three cognitive domains. The psychomotor speed/executive function domain was identical in each study. However, the measures used by Bondi et al. for the language domain were somewhat less correlated than in our study (*r* = 0.27 versus *r* = 0.54). Similarly, the measures used by Bondi et al. for the episodic memory domain were somewhat less correlated than in our study (*r* = 0.54 versus *r* = 0.81). Whilst Bondi et al. (1) did not discuss how ‘statistically dependent’ domain subtests should be, the correlation coefficient for our episodic memory measures (Logical Memory immediate and delayed recall) appears rather high. An implication of this could be that individuals (not) showing impairment on one episodic memory subtest might (not) be impaired on the other. This could result in some of the current patients being misclassified (i.e., SCD_Jak/Bondi_ misclassified as MCI_Jak/Bondi_ or vice versa) due to the two episodic memory subtests measuring similar constructs. Indeed, we calculated the proportion of patients with ‘discordant’ *z*-scores (i.e., for which only one score was ≥1 SD below norms) for each cognitive domain in a combined sample (*n* = 421) of SCD_Jak/Bondi_ and MCI_Jak/Bondi_ (see Table S3 for further details). The proportions of *z*-score discordant patients were: episodic memory domain (*n* = 71; 17%); verbal fluency domain (*n* = 93; 22%); and psychomotor speed/executive function domain (*n* = 108; 26%). A chi-squared test revealed that the difference in these proportions was statistically significant: *Χ*^2^ (*df* = 2, *n* = 421) = 9.7, *p* <0.01. Given the potential for the episodic memory measures used in this study to influence the classification of patients with SCD/MCI, we encourage future researchers to explore the impact of MCI criteria on SCD prognosis using less closely related subtests of episodic memory.

**Table S2.** Ethnicity characteristics of the study samples, as well as the local population served by the memory clinic

| **Sample** | **Total *n*** | **Ethnicity grouping** | | | | |
| --- | --- | --- | --- | --- | --- | --- |
|  |  | **White** | **Asian** | **Mixed** | **Black** | **Other** |
| SCD_Winblad_ | 86 | 82 (97%) | 1 (1%) | 2 (2%) | 0 (0%) | 0 (0%) |
| SCD_Jak/Bondi_ | 185 | 177 (95%) | 5 (3%) | 2 (1%) | 1 (1%) | 0 (0%) |
| UK Census 2011 | 48,430 | 47,268 (98%) | 744 (2%) | 144 (<1%) | 192 (<1%) | 85 (<1%) |
| UK Census 2021 | 56,210 | 55,185 (98%) | 745 (1%) | * | 100 (<1%) | 180 (<1%) |

Data for the study samples is identical to that reported in the main text (see Table 2). Local population data are taken from [2011](https://www.nomisweb.co.uk/census/2011/lc2109ewls) and [2021](https://www.ons.gov.uk/peoplepopulationandcommunity/culturalidentity/ethnicity/datasets/ethnicgroupbyageandsexinenglandandwales) census data publicly available online. The reported census data map to all adults aged 65 and above living in the catchment area of the memory clinic (i.e., Epping Forest, Harlow, and Uttlesford local authorities). *The 2021 census did not report population statistics for the mixed ethnic group, because all data were presented for each specific age separately (e.g., 65 years, 66 years etc), resulting in counts below 10 for the mixed ethnic group. To protect the confidentiality of individuals’ data, cell counts below 10 are suppressed in publicly available census data.

**Table S3.** Correlation statistics for pairs of tests used to operationalize cognitive domains for Jak/Bondi MCI criteria in Bondi et al. (1) and the current study

| **Domain / Study** | | **Tests/test conditions** | **Correlation sample** | **Correlation statistics** |
| --- | --- | --- | --- | --- |
| Language* | Bondi et al. | Category fluency & BNT | Older adults (*n* = 107), including amnestic MCI (*n* = 37), SCD (*n* = 37), and healthy controls (*n* = 33) (4) | *r_Pearson_* = 0.27, *p* <0.01 |
|  | Current study | Category fluency & letter fluency |  | *r_Pearson_* = 0.54, *p* <0.05 |
| Psychomotor speed/ executive function | Bondi et al. | TMT-A & TMT-B | *NA* – correlation statistics not reported here: the same tests were included in this domain by both Bondi et al. and the current study | |
|  | Current study | TMT-A & TMT-B |  |  |
| Episodic Memory | Bondi et al. | RAVLT delayed recall & recognition | Healthy older adults (*n* = 37) (5) | *r_Spearman_* = 0.54, *p* <0.01 |
|  | Current study | LM immediate and delayed recall | Combined sample^#^ (*n* = 421) of patients with SCD_Jak/Bondi_ (*n* = 185) and MCI_Jak/Bondi_ (*n* = 236) taken from the current study sample | *r_Spearman_* = 0.81, *p* <0.01 |

Abbreviations: BNT = Boston Naming Test; TMT-A = Trail Making Test part A; TMT-B = Trail Making Test part B; RAVLT = Rey Auditory Verbal Learning Test; LM = Logical Memory; MCI = Mild cognitive impairment; SCD = Subjective cognitive decline. *Bondi et al. (1) defined a ‘language’ domain; we did not administer BNT in the current study, and we thus defined a ‘verbal fluency’ domain instead. ^#^These individuals correspond to the 185 patients with SCD_Jak/Bondi_ in the main paper, as well as 236 individuals excluded due to having MCI_Jak/Bondi_, but who otherwise fulfilled the same criteria used to select the SCD sample (e.g., aged ≥55 years at baseline; ≥12 months of follow-up; affective symptoms in the clinically normal range).

**Table S4.** The RECORD statement – checklist of items, extended from the STROBE statement, that should be reported in observational studies using routinely collected health data (6)

|  | **Item No.** | **STROBE items** | **Location in manuscript where items are reported** | **RECORD items** | **Location in manuscript where items are reported** |
| --- | --- | --- | --- | --- | --- |
| **Title and abstract** | | | | | |
|  | 1 | (a) Indicate the study’s design with a commonly used term in the title or the abstract (b) Provide in the abstract an informative and balanced summary of what was done and what was found | For 1 (a) and (b) please see Abstract | RECORD 1.1: The type of data used should be specified in the title or abstract. When possible, the name of the databases used should be included.  RECORD 1.2: If applicable, the geographic region and timeframe within which the study took place should be reported in the title or abstract.  RECORD 1.3: If linkage between databases was conducted for the study, this should be clearly stated in the title or abstract. | - 1. – Abstract   2. – Abstract   3. – N/A |
| **Introduction** | | | | | |
| Background rationale | 2 | Explain the scientific background and rationale for the investigation being reported | See Background |  |  |
| Objectives | 3 | State specific objectives, including any prespecified hypotheses | N/A – Study was explorative, comparing the prognostic implications of criteria used to exclude MCI for SCD ascertainment |  |  |
| **Methods** | | | | | |
| Study Design | 4 | Present key elements of study design early in the paper | See *Study design and setting* under Methods |  |  |
| Setting | 5 | Describe the setting, locations, and relevant dates, including periods of recruitment, exposure, follow-up, and data collection | See *Study design and setting* and *Study population* under Methods |  |  |
| Participants | 6 | *(a) Cohort study* - Give the eligibility criteria, and the sources and methods of selection of participants. Describe methods of follow-up  *Case-control study* - Give the eligibility criteria, and the sources and methods of case ascertainment and control selection. Give the rationale for the choice of cases and controls  *Cross-sectional study* - Give the eligibility criteria, and the sources and methods of selection of participants  *(b) Cohort study* - For matched studies, give matching criteria and number of exposed and unexposed  *Case-control study* - For matched studies, give matching criteria and the number of controls per case | (a) *Cohort study* - See *Study design and setting*, *Study population,* and *Derivation of SCD samples* under Methods | RECORD 6.1: The methods of study population selection (such as codes or algorithms used to identify subjects) should be listed in detail. If this is not possible, an explanation should be provided.  RECORD 6.2: Any validation studies of the codes or algorithms used to select the population should be referenced. If validation was conducted for this study and not published elsewhere, detailed methods and results should be provided.  RECORD 6.3: If the study involved linkage of databases, consider use of a flow diagram or other graphical display to demonstrate the data linkage process, including the number of individuals with linked data at each stage. | 6.1 – See *Study population, Diagnosis* and *Derivation of SCD samples* under Methods  6.2 – N/A, reliable diagnostic data were already available  6.3 – N/A, no linkage was performed |
| Variables | 7 | Clearly define all outcomes, exposures, predictors, potential confounders, and effect modifiers. Give diagnostic criteria, if applicable. | See *Study population, Procedures* and *Diagnosis* under Methods | RECORD 7.1: A complete list of codes and algorithms used to classify exposures, outcomes, confounders, and effect modifiers should be provided. If these cannot be reported, an explanation should be provided. | 7.1 – See *Diagnosis* and *Derivation of SCD samples* under Methods |
| Data sources/ measurement | 8 | For each variable of interest, give sources of data and details of methods of assessment (measurement).  Describe comparability of assessment methods if there is more than one group | See *Procedures* and *Measures* under Methods |  |  |
| Bias | 9 | Describe any efforts to address potential sources of bias | No specific biases were identified |  |  |
| Study size | 10 | Explain how the study size was arrived at | Outlined in Figure 1 |  |  |
| Quantitative variables | 11 | Explain how quantitative variables were handled in the analyses. If applicable, describe which groupings were chosen, and why | N/A – primary analysis centered on incidence rates and ratios |  |  |
| Statistical methods | 12 | (a) Describe all statistical methods, including those used to control for confounding  (b) Describe any methods used to examine subgroups and interactions  (c) Explain how missing data were addressed  (d) *Cohort study* - If applicable, explain how loss to follow-up was addressed  *Case-control study* - If applicable, explain how matching of cases and controls was addressed  *Cross-sectional study* - If applicable, describe analytical methods taking account of sampling strategy  (e) Describe any sensitivity analyses | For 12 (a), (b) and (c), see *Statistical analyses* section under Methods  12 (d) is N/A, the same sample was used for both analyses, albeit with a different definition of SCD ‘caseness’. Whilst the patients originally diagnosed with MCI_Winblad_ (some of whom were reclassified as SCD_Jak/Bondi_) were followed up more regularly, the overall amount of follow-up did not differ between samples. |  |  |
| Data access and cleaning methods |  | .. |  | RECORD 12.1: Authors should describe the extent to which the investigators had access to the database population used to create the study population.  RECORD 12.2: Authors should provide information on the data cleaning methods used in the study. | 12.1 – See  *Availability of data and materials* under Declarations  12.2 – See *Data cleaning and missing data* under Results |
| Linkage |  | .. |  | RECORD 12.3: State whether the study included person-level, institutional-level, or other data linkage across two or more databases. The methods of linkage and methods of linkage quality evaluation should be provided. | 12.3 – N/A, A single database was harnessed for this study |
| **Results** | | | | | |
| Participants | 13 | (a) Report the numbers of individuals at each stage of the study (*e.g.*, numbers potentially eligible, examined for eligibility, confirmed eligible, included in the study, completing follow-up, and analysed)  (b) Give reasons for non-participation at each stage.  (c) Consider use of a flow diagram | For 13 (a), (b) and (c), see Figure 1 | RECORD 13.1: Describe in detail the selection of the persons included in the study (*i.e.,* study population selection) including filtering based on data quality, data availability and linkage. The selection of included persons can be described in the text and/or by means of the study flow diagram. | 13.1 – Outlined in Figure 1 |
| Descriptive data | 14 | (a) Give characteristics of study participants (*e.g.*, demographic, clinical, social) and information on exposures and potential confounders  (b) Indicate the number of participants with missing data for each variable of interest  (c) *Cohort study* - summarise follow-up time (*e.g.*, average and total amount) | 14 (a) – see Table 2. For (b), see Table S1. For (c), see Tables 2 and 4. |  |  |
| Outcome data | 15 | *Cohort study* - Report numbers of outcome events or summary measures over time  *Case-control study* - Report numbers in each exposure category, or summary measures of exposure  *Cross-sectional study* - Report numbers of outcome events or summary measures | See Tables 3 and 4. |  |  |
| Main results | 16 | (a) Give unadjusted estimates and, if applicable, confounder-adjusted estimates and their precision (e.g., 95% confidence interval). Make clear which confounders were adjusted for and why they were included  (b) Report category boundaries when continuous variables were categorized  (c) If relevant, consider translating estimates of relative risk into absolute risk for a meaningful time period | See Results and Table 4. |  |  |
| Other analyses | 17 | Report other analyses done—e.g., analyses of subgroups and interactions, and sensitivity analyses | N/A – no other substantive analyses done |  |  |
| **Discussion** | | | | | |
| Key results | 18 | Summarise key results with reference to study objectives | See Discussion section |  |  |
| Limitations | 19 | Discuss limitations of the study, taking into account sources of potential bias or imprecision. Discuss both direction and magnitude of any potential bias | See *Limitations* section in Discussion | RECORD 19.1: Discuss the implications of using data that were not created or collected to answer the specific research question(s). Include discussion of misclassification bias, unmeasured confounding, missing data, and changing eligibility over time, as they pertain to the study being reported. | See *Limitations* section in Discussion |
| Interpretation | 20 | Give a cautious overall interpretation of results considering objectives, limitations, multiplicity of analyses, results from similar studies, and other relevant evidence | See Discussion section, including *Conclusions* |  |  |
| Generalisability | 21 | Discuss the generalisability (external validity) of the study results | See *Strengths* section in Discussion |  |  |
| **Other Information** | | | | | |
| Funding | 22 | Give the source of funding and the role of the funders for the present study and, if applicable, for the original study on which the present article is based | See Declarations |  |  |
| Accessibility of protocol, raw data, and programming code |  | .. |  | RECORD 22.1: Authors should provide information on how to access any supplemental information such as the study protocol, raw data, or programming code. | See *Availability of data and materials* under Declarations |

*Checklist is protected under Creative Commons Attribution ([CC BY](http://creativecommons.org/licenses/by/4.0/)) license.

**References**

1. Bondi MW, Edmonds EC, Jak AJ, Clark LR, Delano-Wood L, McDonald CR, et al. Neuropsychological criteria for mild cognitive impairment improves diagnostic precision, biomarker associations, and progression rates. J Alzheimers Dis. 2014;42(1):275-89.

2. Winblad B, Palmer K, Kivipelto M, Jelic V, Fratiglioni L, Wahlund LO, et al. Mild cognitive impairment--beyond controversies, towards a consensus: report of the International Working Group on Mild Cognitive Impairment. J Intern Med. 2004;256(3):240-6.

3. Petersen RC, Morris JC. Mild cognitive impairment as a clinical entity and treatment target. Arch Neurol. 2005;62(7):1160-3; discussion 7.

4. Nutter-Upham KE, Saykin AJ, Rabin LA, Roth RM, Wishart HA, Pare N, et al. Verbal fluency performance in amnestic MCI and older adults with cognitive complaints. Arch Clin Neuropsychol. 2008;23(3):229-41.

5. Filardi M, Barone R, Bramato G, Nigro S, Tafuri B, Frisullo ME, et al. The Relationship Between Muscle Strength and Cognitive Performance Across Alzheimer's Disease Clinical Continuum. Front Neurol. 2022;13.

6. Benchimol EI, Smeeth L, Guttmann A, Harron K, Moher D, Petersen I, et al. The REporting of studies Conducted using Observational Routinely-collected health Data (RECORD) statement. PLoS Med. 2015;12(10):e1001885.
